# High Energy-Consuming Industrial Transfers and Environmental Pollution in China: A Perspective Based on Environmental Regulation

**DOI:** 10.3390/ijerph182211866

**Published:** 2021-11-12

**Authors:** Shijin Wang, Huiying Zhou

**Affiliations:** Business School, Jiangsu Normal University, Xuzhou 221116, China; zhouhuiying@jsnu.edu.cn

**Keywords:** transfer of high energy-consuming industries, environmental pollution, environmental regulation, dynamic spatial Durbin model

## Abstract

Environmental regulation and industrial restructuring are highly relevant to environmental governance and green development in China. A dynamic game model was used to analyse the mechanisms behind the role of environmental regulation in promoting the restructuring of energy-intensive industries. A dynamic spatial Durbin model was constructed to empirically test the dynamic spatial effects and regional heterogeneity of the transfer of high energy-consuming industries on environmental pollution under environmental regulation with panel data. Thus, the study found that the environmental emission effect of a single high energy-consuming industry transfer and environmental regulation is significant, while the combined effect of both exhibits a negative environmental effect. Environmental regulation and the concentration of high energy-consuming industries have a positive spatial spillover effect on environmental pollution, and the long-term effect is greater than the short-term effect; high energy-consuming industries are gradually transferred from the more developed regions in eastern and central China to the less developed regions in western China.

## 1. Introduction

**Figure 1:**
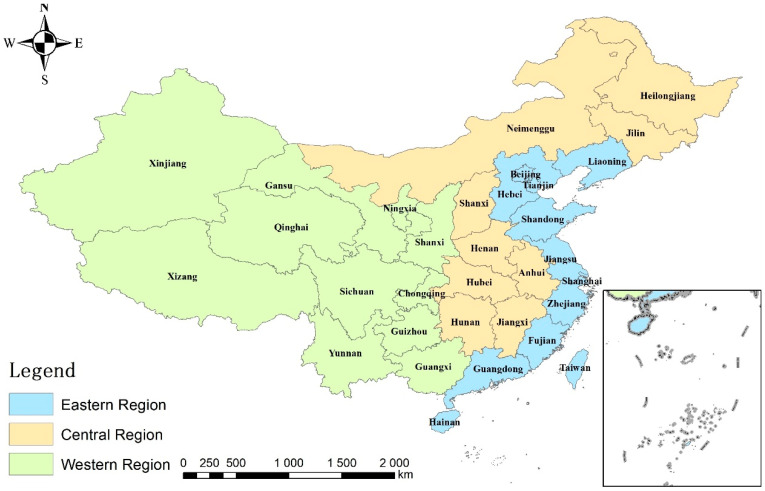
China’s three major regions division map.

After the tax-sharing reform in 1994, the central government gradually transferred economic decision-making power to local governments to stimulate the rapid growth of the local economy. Meanwhile, the performance evaluation mechanism based on economic growth encourages local governments to achieve economic catch-up at all costs. Moreover, one of the typical means toward this end is to attract capital inflows from high energy-consuming industries through competing to lower environmental standards. It is generally believed that high energy-consuming industries have the dual nature of both “large scale capital” and “heavy environmental pollution”; that is, while contributing to economic growth, they also harm energy consumption and environmental quality. Therefore, the contradiction between economic development and the environment is prominent, which arouses the relatively contradictory attitudes of local governments to it. On the one hand, China’s stage of economic development determines its extremely limited financial resources. The desire of local governments to promote economic development through more industrial investment has led to a situation of “bottom-up competition” in the development of environmental regulation standards and the implementation of environmental regulation measures by local governments. Therefore, to achieve better results in local GDP championships, local governments take the initiative to lower environmental regulation standards and attract more business investment. On the other hand, local authorities are concerned about the pollution caused by lower standards. In recent years, environmental events have led to increasing atmospheric pollution, as evidenced by rising average global temperatures, frequent natural disasters and haze, which have resulted in a range of social and political issues. Environmental pollution can also have serious adverse physical and psychological effects on residents. If the local government is obsessed with pursuing political achievements without regard to the physical health of the residents and the quality of the living environment, the residents will become distrustful of the government due to this psychological imbalance—the credibility of the government is greatly damaged. In the context of building an ecologically civilized society, the central government and local governments in China are paying increasing attention to environmental protection issues, while the quality and efficiency of development are improving, and regional development is becoming more coordinated; environmental issues have become one of the key factors in assessing the performance of local governments. Thus, under a more stringent assessment system, does the “bottom-up competition” between local governments in environmental regulation still exist in line with the “pollution sanctuary” hypothesis for investment decisions in energy-intensive industries? In the paper, the situation in China is examined: the country is divided into three major regions, the east, the central and the west, each with its distinct social characteristics, as shown in Figure 1. Eastern China has gentle topography, good conditions for agricultural generation and abundant resources such as aquatic products, oil, iron ore and salt. Due to its long history of development, the region is strategically located, the labour force is highly educated and technically strong, and the region has a strong industrial and agricultural base, which plays a leading role in its overall economic development. Central China is located inland and is a food production base with numerous plains. It is rich in energy and various metal and non-metal mineral resources and has a good foundation for heavy industry. Western China is vast, with high terrain and complex topography that is not conducive to crop growth, as most of it is alpine and water-scarce. Due to its late history of development, its economic development and technical management levels are different from the east and central, but the region is also large, rich in mineral resources and has great potential for development.

The relationship between environmental regulation, inter-regional transfer of energy-intensive industries and environmental pollution has received extensive attention from the academic community in both theoretical and empirical studies. Environmental regulation is a form of constraint which restricts individuals or organizations through tangible institutions or intangible awareness to protect the environment. A closely related theory to the link between environmental regulation and industrial restructuring is the “pollution sanctuary” hypothesis [1], which states that highly polluting firms tend to move from areas with strict environmental regulations to areas with lax environmental regulations, making them a pollution sanctuary. Scholars both in China and abroad have mainly held two different views on this hypothesis. One side believes that there is a significant correlation between environmental regulations and the relocation of high energy-consuming industries, and that there is a pollution sanctuary effect in areas with weaker environmental regulations. It was suggested that high energy-consuming industries seek cost advantages in relocating to areas with lax environmental regulations so that the location becomes a sanctuary for pollution transfer [2]. In contrast, domestic studies have focused on the question of whether high pollution industries in developed countries perceive China as a pollution sanctuary. Scholars’ research indicates that interregional “pollution sanctuary” effects—groups of polluting industries that migrate to an area with low environmental regulation—exist within China, and that there is a clear trend of such shifts from developed regions in eastern China to less developed regions in the central and western regions, and from environmentally sensitive regions to non-environmentally sensitive regions [3,4,5]. Moreover, the spatial distribution of the high-pollution industries within China’s developed regions has been undergoing a dramatic restructuring, demonstrating a tendency to switch from concentration to dispersion, with the geographical distribution characterized by a “concentration in the centre to dispersion in the periphery” [6]. On the other hand, the pollution sanctuary hypothesis may not be valid as there was no significant correlation found between environmental pollution and the locational adjustment of highly polluting enterprises. This hypothesis has been challenged in the literature through reviews of earlier studies that did not provide sufficient evidence for the presence of so-called pollution sanctuary effects [7,8]. Explanations were obtained from theoretical perspectives regarding environmental costs, industrial structure and locational decisions [9,10]. Additionally, empirical studies revealed that the main reason why the empirical results do not support the pollution sanctuary effect is the endogeneity of environmental regulatory cost variables [11].

Regarding the study of the environmental effects of the transfer of high energy-consuming industries, the relevant literature has the following three main findings. Firstly, the transfer of energy-intensive industries had a pollution agglomeration effect, which exacerbates environmental pollution in the receiving region. Most scholars’ empirical studies showed that the agglomeration induced by industrial transfer has an environmental pollution effect and, specifically, industrial transfer deepens the spatial link between the economy and pollution [12,13]. It was believed that spatial spillover effects of transfer and pollution, where a behavior not only has an impact on its region but also on other regions, cause greening adjustments in the industrial structure of the source region and deteriorating developments in less-developed adjacent regions [14,15]. In the context of China’s specific situation, it was found that the western region of China is the primary zone that bears the shift of pollution that has contributed to increased pollution levels in the region [16]. Additionally, the relocation of high energy-consuming industries—including traditional manufacturing, agricultural resource-based industries and heavy chemical industries—has exacerbated energy consumption and pollution emissions in the receiving region, while yielding a positive effect of energy saving and emission reduction in the transferring region [17]. Secondly, the transfer of high energy-consuming industries had the effect of driving a pollution reduction, which in turn improved the environmental pollution situation in the receiving region. It was found that the industrial transfer in each investigation period significantly diminished the total industrial wastewater discharge in the Yangtze River Economic Zone on the whole. The reason for this may be that the pollutant emissions are higher than the maximum permitted under the level of environmental regulation in the transferring site, which is relatively strict [18]. At the same time, barriers to exit exist for enterprises in pollution-transferring areas, which help to mitigate pollution transfer effects [15,19]. In addition, the agglomeration effect and technological knowledge spillover effect of the increased share of high energy-consuming industries resulted in scoped economies of transfer, allowing these enterprises to reveal larger positive externalities in energy saving and emission reduction, thus cutting pollution severity [20,21]. Finally, the environmental effects of the transfer of energy-intensive industries did not have a single positive or negative effect but presented a non-linear relationship. It was concluded that the impact of industrial agglomeration on environmental pollution is gradual, uncertain and alternating, as environmental quality might deteriorate or improve under different levels of environmental regulation [22]. The study proved that the relationship between the transfer of high energy-consuming industries and environmental pollution exhibits a gradually increasing non-linear relationship: as the relative intensity of environmental regulation gradually shifts from low to high thresholds, the environmental pollution problems caused by the transfer of high energy-consuming industries become more serious [23]. Furthermore, scholars have found a significant single threshold effect on the relationship between industrial agglomeration and air pollution, using the technological innovation capacity of air pollution prevention and control as the threshold variable [24].

Comprehensive domestic and international research indicated that studies on the impact of regional industrial transfers on environmental pollution have focused on the impact of foreign direct investment on the environment, concentrating on the existence of polluted sanctuaries in China. However, the issues of the adaptation of an industrial structure to its internal resources and environment remain prominent, and relatively few studies have been conducted on the environmental effects of interregional transfers of high energy-consuming industries in China. Existing studies mainly investigate the correlation between industrial relocation and environmental pollution [25], whereas environmental regulation influences the inter-regional relocation of high energy-consuming industries, which in turn has an impact on environmental pollution in China. Therefore, the intrinsic links among the three, especially the moderating role of environmental regulation, deserve further exploration. Additionally, in terms of empirical studies, scholars have mostly considered the spatial effects of such impacts but from a static spatial model only, lacking consideration of dynamic effects [26]. Thus, from the perspective of environmental regulation, this paper analyses the mechanism of interaction between environmental regulation and structural changes in energy-intensive industries with a dynamic game-based model. Secondly, the dynamic spatial Durbin model is used to empirically analyse the impact of environmental regulation tools and energy-intensive industrial shifts on the environment, and to further examine the relationship sub-regionally. Finally, operational policy recommendations are proposed to improve the ecological environment and promote the transformation and upgrading of high energy-consumption industries.

## 2. Game Modelling

Environmental regulation policies, on the one hand, guide producers’ production behaviour as well as structural adjustment decisions by changing firms’ explicit or implicit costs. On the other hand, different policy designs create different incentives for the behaviour of economic agents, thus influencing the trend of industrial shifts [27]. Therefore, a dynamic game model is developed to specifically analyse these relationships’ internal mechanisms of action. The dynamic game model between local governments and energy-intensive enterprises is shown below. It focuses on the impacts of environmental regulation and competition by local governments on the spatial composition and layout of high energy-consuming industries, and how the environmental policy choices of local governments have a significant impact on the game dynamics. The model assumptions are as follows.

(1) It is assumed that the high energy-consuming enterprise is *A* and the region is $R_{1}$ and $R_{2}$, where the enterprise is located in the region $R_{1}$.
(2) High energy-consuming enterprise A, if in the violation of environmental regulations and related policies the enterprise still does not take action to obtain the benefits of not taking measures for x, if the cost of taking measures for y, and if it additionally brings social benefits for z.
(3) It is assumed that region $R_{1}$. discovers an environmental violation in enterprise A and imposes a fine of p, incurring a monitoring cost of s.
(4) Suppose that region $R_{1}$ imposes a penalty on the enterprise A. The loss caused by the breach is $l_{1}$, the economic loss to the region itself is $l_{2}$, and the additional cost of governance due to the breach is e.
(5) Assume that the regional $R_{1}$ and regional $R_{2}$ subsidies for high energy-consuming enterprises using environmental instruments are $g_{1}$ and $g_{2}$ respectively.
(6) The cost of transferring the two regions is t and the expected benefit of transferring to the new region $R_{2}$ is r.

**Figure 2:**
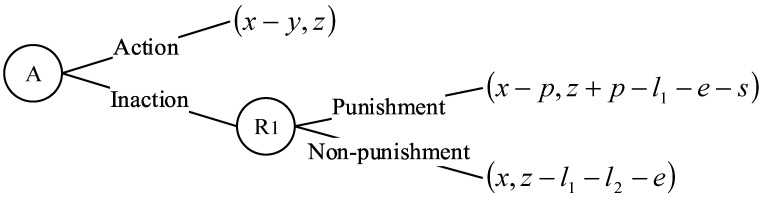
The gaming process in scenario 1.

**Figure 3:**
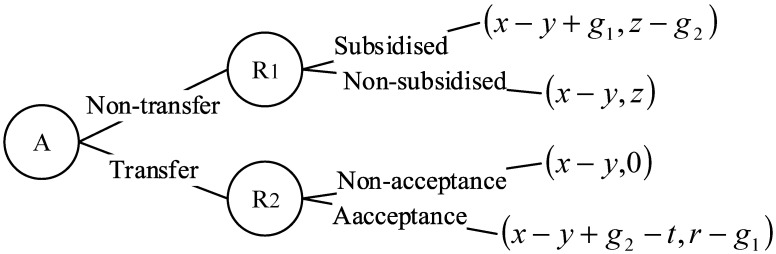
The gaming process in scenario 2.

This game model involves a three-party equilibrium of interests: the enterprise, the region and the society. In the three-party game process, the choice of action of each game party affects the policy decisions of the other game parties, and the dynamic game shown in Figure 2 and Figure 3 were made in the order of decision making.

Scenario 1 describes the change in the interests of the three parties if high-polluting enterprise *A* does not temporarily undergo industrial relocation behaviour and adopts certain environmental protection behaviour in the region.

Figure 2 showed that if $z+p-l_{1}-e-s>z-l_{1}-l_{2}-e$, namely $p+l_{2}>s$, then $R_{1}$ chooses to penalize. In response to the penalty, firm *A* has two types of behaviour: action and inaction. The comparison shows that when x−p<x−y, namely y<p, then firm *A* will act to take precautionary measures to protect the environment, but this is a short-term equilibrium. As firms pay for energy saving and emission reduction, especially when the government implements stricter environmental policies, it will make firm *A* spend more on equipment and fixed assets to deal with the environment and invest in related factors. In this case, it is necessary to compare y and t. If y<t, enterprise *A* will continue to take action in the region $R_{1}$ to avoid polluting behaviour; if y>t the regional transfer of industry begins to appear, and thus the regional transfer of high energy-consuming enterprises is the best choice for transferring risk.

Scenario 2 describes the change in the three-party benefits of whether subsidies are given to different regions $R_{1}$ and $R_{2}$ if industrial transfer behaviour occurs in high polluting firm *A*.

It can be seen from Figure 3 that the region $R_{2}$ is faced with the decision of whether to accept the transfer of enterprise *A* or not. The comparison shows that when $r-g_{1}>0$, namely $r>g_{1}$, $R_{2}$ will allow enterprise *A* to move in. Therefore $R_{1}$, which implements strict environmental regulations, will choose to give enterprise *A* appropriate government subsidies to retain enterprise *A* in the region, at which time enterprise *A* can choose to transfer or not. When $x-y+g_{2}-t<x-y+g_{1}$, namely $g_{2}<t+g_{1}$, firm *A* will choose not to transfer; when $g_{2}>t+g_{1}$, firm *A* will choose to transfer.

The dynamic game described above revealed that the selection of environmental regulation policies in a given region influences the environmental decisions of the linked region. Consequently, local governments need to assess the benefits of high energy-consuming enterprises versus the costs of environmental pollution, as well as focusing on the environmental policies of the relevant local governments, in order to make trade-offs between the regional relocation of high energy-consuming enterprises and economic development.

## 3. Materials and Methods

### 3.1. Model Setting

The paper draws on the spatial Durbin panel model [28], which is a powerful tool for analyzing the spatial effects of environmental pollution, to discuss the effects of industrial transfer and environmental regulation on environmental pollution in both temporal and spatial terms. Hence, the geographical distance matrix (which is set according to the inverse of the geographical distance between two regions) is chosen according to the spatial autocorrelation test to build a dynamic spatial Durbin model to investigate the spatial effects of environmental regulation, as well as the transfer and agglomeration of high energy-consuming industries, on environmental pollution.

#### 3.1.1. Spatial Correlation Test

Spatial autocorrelation analysis, which essentially examines the spatial dependence of the dependent variable, determines whether the spatial measure is effective with testing the spatial autocorrelation of the dependent variable. Therefore, global spatial autocorrelation analysis provides an analytical method for measuring the overall spatial differences between different regions as well as the degree of spatial correlation. The global Moran’s I index is generally used to measure the general characteristics of environmental pollution correlation with the following formula:

(1) $$I=\frac{\sum_{i=1}^{n}\sum_{j=1}^{n}w_{ij}\left(x_{i}-\bar{x}\right)\left(x_{j}-\bar{x}\right)}{S^{2}\sum_{i=1}^{n}\sum_{j=1}^{n}w_{ij}}$$

where $S^{2}=\frac{\overset{n}{\underset{i=1}{\sum }}{\left(x_{i}-\bar{x}\right)}^{2}}{n}$ represents sample variance, *n* is the number of sample areas, *w_ij_* is the element of the spatial weight matrix (*i*, *j*) (used to measure the distance between area *i* and area *j*), and *x_i_* and *x_j_* are the observation values of area *i* and *j* respectively; $\bar{x}$ is the average of the observations.

Local spatial autocorrelation analysis is available to analyse whether local regional agglomerations are similar or dissimilar observations. Additionally, the local Moran’s I index is used to test for spatial agglomerations in the vicinity of a particular region, with the formula being:

(2) $$I=\frac{(x_{i}-\bar{x})\sum_{i=1}^{n}\sum_{j=1}^{n}W_{ij}{\left(x_{i}-x_{j}\right)}^{2}}{S^{2}}$$

where it is shown that a positive Moran’s I index indicates that high (low) values in the region are surrounded by neighbouring high (low) values; a negative value indicates that high (low) values in the region are surrounded by neighbouring low (high) values. The scatter plot of Moran’s I index, derived from the local correlation analysis, shows the local spatial correlation of environmental pollution.

#### 3.1.2. Spatial Durbin Panel Model

The spatial econometric models are the spatial lag model (SAR), which is defined as the influence of the dependent variable acting on other spatial regions through a spatial transmission mechanism; the spatial error model (SEM), in which random errors are spatially correlated; and the spatial Durbin model (SDM), which combines the characteristics of SAR and SEM by introducing lagged terms of the independent variable and dependent variable to accurately estimate spatial effects through considering the effects of appropriate spatial structure [29]. Thus, the spatial effects Durbin model is formulated as that,

(3) $$EPI_{it}=\alpha I_{n}+\rho WEPI_{it}+\beta X_{it}+\theta WX_{it}+\gamma_{t}I_{n}+\mu_{i}+\varepsilon_{it}$$

where $EPI_{it}$ is the environmental pollution composite index for region *i* in year *t*, $X_{it}$ is the matrix of exogenous explanatory variables of order n × k (k is the number of explanatory variables). Additionally, *I_n_* is the n × 1 unit vector, *W* is the matrix of spatial weights of order n × n, *ρ* is the spatial autoregressive coefficient taking values in the range [−1, 1], *β* is the vector of location parameters to be estimated and *θ* is the vector of corresponding impact coefficients. $WEPI_{it}$ and $WX_{it}$ represent endogenous interaction effects between the explained variables and exogenous interaction effects between the explanatory variables, respectively. $\gamma_{t}$ and $\mu_{i}$ denote temporal and individual effects, respectively. $\varepsilon_{it}$ is the random error term.

The above model is a static spatial effects model, hardly solving the problems of certain unobservable spatial effects, certain temporal effects, the existence of endogeneity of explanatory variables, etc. Accordingly, the paper adds a consideration of dynamic effects and constructs a dynamic spatial Durbin model, as shown in Equation (4).

(4) $$EPI_{it}=\tau EPI_{it-1}+\rho WEPI_{it}+\eta WEPI_{it-1}+\beta X_{it}+\theta WX_{it}+\gamma_{t}I_{n}+\mu_{i}+\varepsilon_{it}$$

where $EPI_{it-1}$ is the first-order lag term of the *EPI*, τ is the corresponding coefficient, $\tau EPI_{it-1}$ the temporal-lag term of the *EPI* and $\eta WEPI_{it-1}$ represents the space-temporal lag term of the comprehensive environmental pollution index.

#### 3.1.3. Direct Effect and Indirect Effect

Many empirical studies have used point estimates from one or more spatial regression models to verify the existence of spatial spillover effects. However, using point estimates to test is biased and changing variables in different models affects the partial differential equation. Therefore, to estimate accurately how the explanatory variables affect the explained variables, the paper uses the method of Elhorst to decompose the effect of the explanatory variables into a direct effect and an indirect effect. Equation (4) can be rewritten as follows:

(5) $$\begin{array}{l} EPI_{it}={\left(I_{n}-\rho W\right)}^{-1}\left(\tau I_{n}+\eta W\right)EPI_{it-1}+{\left(I_{n}-\rho W\right)}^{-1}\left(\beta X_{it}+\theta WX_{it}\right)\\ +\left(I_{n}-\rho W\right)\left(\gamma_{t}I_{n}+\mu_{i}\right)+{\left(I_{n}-\rho W\right)}^{-1}\varepsilon_{it} \end{array}$$

For the *k*th explanatory variable X from time unit 1 to unit n, the corresponding partial differential matrix of Y is that,

(6) $$\left[\frac{\partial EPI}{\partial x_{1k}},\ldots ,\frac{\partial EPI}{\partial x_{nk}}\right]={\left(I_{n}-\rho W\right)}^{-1}\left[\left.\beta_{k}I_{n}+\theta_{k}W\right]\right.$$

(7) $$\left[\frac{\partial EPI}{\partial x_{1k}},\ldots ,\frac{\partial EPI}{\partial x_{nk}}\right]={\left[\left.\left(1-\tau \right)I_{n}-\left(\rho +\eta \right)W\right]\right.}^{-1}\left[\left.\beta_{k}I_{n}+\theta_{k}W\right]\right.$$

Equations (6) and (7) measure the short-term and long-term spatial effects of the described variables (the explanatory variables), respectively. The direct effect is for each main diagonal element of the partial differential matrix, which manifests itself as a change in a particular explanatory variable for a particular unit, thus changing the explanatory variable for the particular unit itself. Each non-diagonal element represents an indirect effect, meaning that when a particular explanatory variable is changed in a particular unit, the explanatory variables in other units change similarly.

### 3.2. Variable Selection and Data Description

Comprehensive Environmental Pollution Index (*EPI*). Environmental pollution in China mainly originates from three industrial wastes (industrial waste gas, industrial wastewater and industrial solid waste). As the statistical unit of industrial waste gas is not consistent with the other two pollutants, and as sulphur dioxide is the main emission in China’s industrial production process according to data from the 2018 Environmental Statistics Yearbook, this paper selected three indicators—industrial sulphur dioxide emissions, industrial waste water emissions and industrial solid waste emissions markers—to construct an *EPI* measure of the general level of environmental pollution in each region [22,30]. The larger the Environmental Pollution Index value, the more serious the environmental pollution or the worse the environmental conditions in the region, and conversely, the better the environmental pollution in the region.

(8) $$EPI_{it}=WP_{it}/\sum WP+AP_{it}/\sum AP+SP_{it}/\sum SP$$

where $WP_{it}$, $AP_{it}$ and $SP_{it}$ are industrial wastewater emissions, industrial sulphur dioxide emissions and industrial solid waste generation in region *i* in year t, respectively. ∑WP, ∑AP and ∑SP are the total emissions of industrial wastewater, industrial sulphur dioxide and industrial solid waste in year t, respectively.

High energy-consuming industries transfer (*EIT*). Regarding the measurement of regional industrial transfer, the majority of alternative indicators are used to measure industrial transfer at the inter-provincial level, but the existing research has not yet reached a consensus on the specific selection of indicators [12,31,32]. The existing metrics are mainly of three types, including the use of relevant share indicators reflecting the indirect amount of interregional industrial transfer, interregional input-output tables and deviation share analysis. The inter-regional transfer of polluting industries can be measured by the ratio of the output value of polluting industries in each province to the total national output value of the industry; that is, the concentration of energy-intensive industries, which reflects the transfer of high energy-consuming industry into or out of a region [33]. The ratio of the regional high-energy consumption industry output value to the national total output value is used in this paper to denote the regional high-energy consumption industry market concentration. The economic implication is that the larger the value (*EIT*), the higher the proportion of high energy-consuming industries in the region to the country as a whole, which suggests an accumulation of pollution or a shift of energy-intensive industries into the region. Conversely, it means that the proportion of energy-intensive industries in the country is decreasing and that the region is either decreasing in pollution or moving out of the country.

(9) $$EIT_{ij}=\sum_{j}\left(Q_{ij}/Q_{j}\right)/j$$

where i and j denote region i and high energy-consuming industry j, respectively. *Q_ij_* is the industrial output value of high energy-consuming industry j in the region, and *Qj* represents the national total output value of high energy-consuming industry j. Based on the 2010 National Economic and Social Development Statistics Report, this paper explores six major high energy-consuming industries: chemical raw materials and chemical products manufacturing; non-metallic mineral products; ferrous metal smelting and rolling processing; non-ferrous metal smelting and rolling processing; petroleum processing, coking and nuclear fuel processing; and electricity, heat production and supply.

Environmental Regulation (*ER*). There are four main approaches to assessing environmental regulation: firstly, using specific environmental regulation policy indicators to characterise the effectiveness of pollution control to measure the strength of environmental regulation; secondly, selecting multiple indicators and creating a system of indicators to assess the level of environmental regulation; thirdly, endogenising environmental regulation with income per capita, air circulation coefficient, etc. as indicators; and fourthly, using numerical assignment based on specific criteria to characterise the intensity. This paper constructs environmental regulation proxy variables from the perspective of environmental awareness of polluting subjects [34]. Performance-based environmental regulation (*PER*) is measured by first standardising the three types of pollution emissions data using a mean-standardised approach, and by assigning individual pollution emission weights to each region to more accurately reflect its level of pollution control, as the proportion of industrial emissions, industrial sulphur dioxide and industrial solid waste varies between regions.

(10) $$\left\{\begin{array}{l} w_{ij}=\frac{p_{ij}}{\sum_{i}p_{ij}}/\frac{GDP_{ij}}{\sum_{i}gdp_{i}}\\ PER=1/\left(\frac{1}{3}\sum_{j=1}^{3}w_{ij}P_{ij}\right) \end{array}\right.$$

where $GDP_{i}$ is the gross production value of region i; $p_{ij}$ is the emission of pollutant j in region i; and *P* is the standard value of pollutant emissions. *PER* represents the performance-based environmental regulation intensity, where the lower the value, the lower the environmental regulation intensity, and vice versa.

Another form of environmental regulation is cost-based environmental regulation (*CER*). This paper measures the intensity of cost-based environmental regulation using the share of investment in industrial pollution control projects in industrial value-added, which is set to test the robustness of the empirical results by replacing the core variables.

This article also adds some control variables to ensure the stability of the test results. First (1), the use of GDP per capita (*PGDP*) in each province as a proxy variable for economic development (*ECD*), and the introduction of the squared term of *PGDP* in the model to verify the existence of the Environmental Kuznets Curve (EKC), that is, as the degree of economic development rises, the level of environmental pollution increases first, and after reaching a certain maximum level of pollution, the level of environmental pollution decreases slowly as the economy continues to grow. This shows the existence of an inverted “U” shaped non-linear relationship between economic development and pollution emissions. Second (2), fiscal decentralization (*FIS*) is an indicator to measure the discretionary power of local governments. It is found that the fiscal decentralisation system is an essential variable affecting energy consumption and energy-saving [35], and therefore the ratio of fiscal expenditure to total fiscal expenditure by provinces and municipalities is used to measure the role of fiscal decentralization. Third (3), trade liberalisation allows developed countries to shift pollution-intensive enterprises to use developing countries to make them pollution havens [36]. Therefore, the ratio of total imports and exports to GDP for each province is used to measure the level of trade openness (*OPT*), and the total imports and exports are converted using the current annual US dollar and RMB median prices. Fourth (4), areas with high traffic density produce more pollution, hence the regional transport conditions (*TRA*) are measured as the sum of road and rail mileage operated by each province. Fifth (5), the ratio of the number of students enrolled in higher education to the value of the total population of the region is used as an indirect indicator of the level of human capital (*HUM*), as regions with higher levels of labour are usually those with better economic development. They are more environmentally sustainable and thus likely to improve environmental pollution in the region.

In the paper, a data sample of 30 provinces and cities in China (excluding Tibet, Hong Kong, Macao and Taiwan) was selected for the period 2003–2016, with missing values for individual years filled in by interpolation, and the raw data used were taken from the China Statistical Yearbook, the China Industrial Statistical Yearbook, the China Environmental Yearbook and the Statistical Yearbooks of each province in previous years. Table 1 shows the descriptive statistical information for the main variables.

## 4. Results

### 4.1. Spatial Correlation Analysis

This paper first calculated the Moran’s I index for the dependent and core independent variables from 2003 to 2016, and the results were shown in Table 2. From Table 2, it can be seen that the Moran’s I index for environmental pollution, transfer of high energy consumption industries and environmental regulation are all positive, and they all pass the statistical significance level at each time point, which indicates that all four variables demonstrate positive spatial correlation with spatial clustering characteristics.

**Figure 4:**
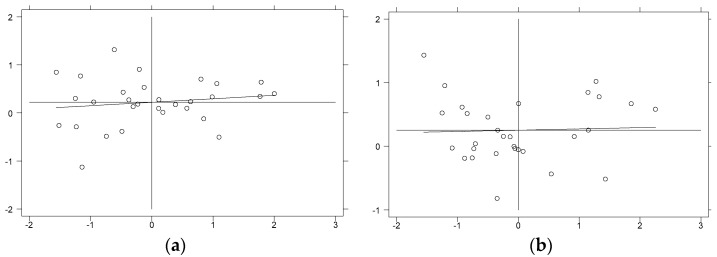
Local Moran’s I scatter plot of environmental pollution. (**a**) Year of 2003. (**b**) Year of 2016. Note: The top left corner of the image is the first quadrant, then counterclockwise in order, the second, third and fourth quadrants.

**Figure 5:**
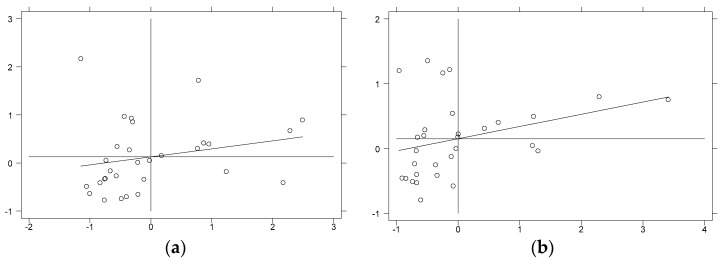
Local Moran’s I scatter diagram of the transfer of high energy-consuming industries. (**a**) Year of 2003. (**b**) Year of 2016. Note: The top left corner of the image is the first quadrant, then counterclockwise in order, the second, third and fourth quadrants.

Local spatial autocorrelation tests were conducted using local Moran’s I indices for environmental pollution in 30 provinces and cities in China from 2003 to 2016, and scatter plots of Moran’s I indices for representative years were plotted. Figure 4 showed the scatter plots of Moran’s I index for the distribution of regional environmental pollution in China in 2003 and 2016, and Figure 5 showed the scatter plots of Moran’s I index for the distribution of regional energy-intensive industrial transfers in China in 2003 and 2016. Specifically, the first and third quadrants indicate that high values are correlated with high values and low values are correlated with low values for specific regions and adjacent regions, respectively, namely the existence of positive spatial autocorrelation; the second and fourth quadrants represent that low values are correlated with high values and high values are correlated with low values for specific regions and adjacent regions, respectively, namely the existence of negative spatial autocorrelation. It is evident from Figure 4 and Figure 5 that the Moran’s I values for environmental pollution and industrial transfer were mostly distributed in the first and third quadrants in both 2003 and 2016, showing that the levels of environmental pollution and industrial transfer in most regions of China are positively correlated with the surrounding areas.

### 4.2. Spatial Econometric Model Empirical Results

The appropriate spatial econometric model was selected based on Moran’s I test. Firstly, the LM test was performed, with the results of LM-Lag, LM-Error, robust LM-Lag and robust LM-Error all significant at the 5% level, stating that the spatial econometric model is applicable to the paper. Secondly, the results of the LR likelihood ratio test indicated that the statistics pass the significance test at the 5% level of significance, which means that the spatial Durbin model (SDM) that is more suitable for fitting the data in the paper can be simplified to a spatial lagged model (SLM) and a spatial error model (SEM). Finally, the Hausman statistic was positive and passed the 5% significance level, signifying that the null hypothesis of random effects needs to be rejected and a fixed-effects model chosen. In summary, the fixed-effects spatial Durbin model should be adopted for the analysis of the spatial effects in this paper. The test results were shown in Table 3.

Considering the influence of dynamic effects, the paper added the time-lagged and space-time-lagged conditions of environmental pollution from the perspective of the fixed-effects to construct a dynamic spatial Durbin model. Meanwhile, to ensure the robustness of the study, both the static Durbin model and the ordinary panel regression model were used to compare with it, and Table 4 showed the regression results of these three models. Model (1) is a dual fixed dynamic spatial Durbin model with time-lagged and space-time-lagged terms for both individual and time effects, and model (2) is a dynamic spatial Durbin model with fixed time effects for the space-time lagged terms; models (3) and (4) are static spatial Durbin models with fixed effects while models (5) and (6) are panel regression models with fixed effects. From Table 4, it can be seen that the spatial autoregressive coefficients ρ in the dynamic spatial Durbin model all pass the 1% significance level, while coefficients ρ in the static spatial Durbin model are less significant than those in the dynamic spatial Durbin model. This suggests that environmental pollution has a significant spatial spillover effect; in other words, an increase in the level of local environmental pollution leads to a deterioration in the environmental pollution in neighbouring regions. Simultaneously, the significance of the estimated coefficients of both the dynamic spatial Durbin model and the spatial lag term is higher than that of the static spatial Durbin model and the ordinary panel regression, which shows that the spatial effects consider dynamic effects when studying the transfer of energy-intensive industries and environmental regulation on environmental pollution. Further comparing the R^2^ values of the models, it can be found that the dynamic spatial Durbin model has a higher R^2^ value and its fitting superiority is thus significantly better than the other models. Therefore, it is reasonable to choose the dynamic spatial Durbin method in this paper for focusing on the results of model (1) and model (2).

Firstly, the effect of energy-intensive industry transfer on environmental pollution was studied. Both models have significantly positive coefficients for the transfer of high energy-consuming industries, showing that the agglomeration of polluting industries caused by the transfer of high energy-consuming industries aggravates the environmental pollution in the receiving areas. To promote the transformation and upgrading of local industries and the elimination of outdated production technologies and facilities, industries transferring out of the region transfer industries or production links that are highly polluting and at the relatively low-end of the industrial chain. The transfer of these energy-intensive industries is accompanied by pollution spillover effects. The other side of the coin is that, to fulfil the GDP growth effect of the industrial sector, regions actively undertake industries with high energy consumption and environmental pollution, which aggravates environmental pollution there. The effect of the intensity of environmental regulation is then examined, with regression coefficients of 0.017 and 0.011 for performance-based and cost-based environmental regulation, respectively, with both passing the 1% level of significance. This suggests a positive relationship between environmental regulation and environmental pollution, one reason being that industries with high energy consumption and pollution are clustered in areas with relatively high levels of environmental regulation. This laterally reflects that high energy-consuming industries have not moved to areas with a lower intensity of environmental regulation, which proves that the pollution sanctuary effect does not exist in China [22]. Another possible reason is that with the transfer of polluting industries to improve environmental regulations, local governments increase their investment in pollution control while taking over the transfer of high energy-consuming industries. However, during this process, due to reasons of policy response time and the speed of industrial transfer, a time gap exists between the pollution reduction effect of environmental regulations and the pollution agglomeration effect of high energy-consuming industries, and thus the pollution increase effect is significant in the short term. Further, while examining the coefficients of the cross-products of industrial transfer and environmental regulation, it can be found that the coefficient values are −1.053 and −0.011, respectively, which are significant at least at the 5% level of significance. These indicate that the combination of high energy-consuming industrial transfer and environmental regulation significantly reduces environmental pollution, which indicates that the increase in the level of environmental regulation leads to a negative effect of pollution emissions from the transfer of high energy-consuming industries. Finally, in terms of the control variables, the primary coefficient on GDP per capita (ECD) is positive and the secondary coefficient is negative, reflecting that the fitted curve between environmental pollution and economic growth takes on a more significant inverted “U” shape, which proves the validity of the EKC hypothesis. The coefficient of transport (TRA) is estimated to be significantly positive, indicating that the accessibility of transport has become an important indicator for the local government’s business environment and that the accessibility and improvement of transport can attract more investment enterprises. The transport situation is, therefore, an important factor contributing to environmental pollution. Additionally, fiscal decentralization (FIS), trade openness (OPT) and human capital (HUM) harm environmental pollution, with HUM having the most significant impact. The higher the cost and quality of labour, the more developed the region’s economy generally is and the more advanced its regional awareness of environmental governance and green production processes. Along with awareness of pollution treatment facilities, these factors bring about low levels of environmental pollution.

### 4.3. Analysis of Spatial Effect Estimation Results

According to the partial derivative matrix of Equations (6) and (7), the dynamic spatial Durbin model is decomposed to test the spatial effects of environmental regulation and industrial transfer on environmental pollution. For comparative analysis, this paper also decomposes the effects of the static spatial Durbin model, which, unlike the dynamic spatial Durbin model, does not have short-term effects on the explanatory variables, and the decomposition results were shown in Table 5. Performance-based environmental regulation is used as an example for this paper.

The results of the direct effects show that the agglomeration of high energy-consuming industries and environmental regulations have negative environmental effects and exacerbate environmental pollution. Under the constraints of environmental regulations, the agglomeration of high energy-consuming industries displays positive environmental effects and suppresses environmental pollution, which is consistent with the analysis above. The dynamic spatial Durbin model considers both short-term and long-term direct effects, but the presence of cumulative effects makes the long-term effects greater than the short-term effects. When environmental pollution in the region improves significantly, individuals raise expectations and have higher demands on the environment, leading to government action to regulate it. Moreover, the time-lag between the concentration of high energy-consuming industries and the impact of environmental regulation would have an impact on environmental pollution in the long term. In addition, the long-term direct effects in the spatial Durbin model are larger than those in the static spatial Durbin model, indicating that the static spatial model underestimates the long-term effects.

The indirect effect reflects the influence of the explanatory variables of the neighbouring regions on the explanatory variables of the region, namely the influence of the transfer of high energy-consuming industries in the neighbouring regions and environmental regulations on the environmental pollution in the region. The estimated coefficients of the lagged term of high energy-consuming industry agglomeration are significantly positive, mainly showing that the agglomeration of energy-intensive industries in neighbouring regions has a poverty-inducing effect on the agglomeration of energy-intensive industries in the region, and the region becomes an in-migration area. Therefore, the excessive agglomeration of energy-intensive industries aggravates the environmental pollution in the region. This is due to the presence of a large number of highly polluting enterprises in the neighbouring areas, while the influence of geographical location factors inevitably causes industrial pollutants to spread to the region, resulting in increased pollution in the region. Moreover, resulting from the agglomeration effect of industries, spatially adjacent areas become the primary choice for industrial relocation and pollution radiation. Concerning the indirect effect of environmental regulations, the more stringent environmental regulations in the neighbouring regions aggravate the pollution in the region, because the increase in environmental standards and environmental policies in the neighbouring regions force the enterprises in the region to shift production to the surrounding areas where the environmental costs are lower. Overall, the effect of environmental regulations on pollution emissions is manifested as a positive spatial spillover effect.

### 4.4. Regional Heterogeneity Analysis

According to the results of the dynamic spatial Durbin model, the pollution sanctuary hypothesis does not exist in China. In terms of performance-based environmental regulation, environmental pollution decreases as environmental regulation increases: economic development has an inverted U-shaped relationship with environmental pollution; transport has an overall positive effect on environmental pollution; and fiscal decentralisation, trade openness and human capital hurt environmental pollution. To study the differences in the impact of high energy-consuming industries’ transfer and environmental regulations on environmental pollution in different regions, this paper takes into account the significant regional heterogeneity of environmental pollution, energy-consuming industries’ agglomeration and environmental regulations and select samples from different regions for empirical analysis. According to the classification criteria on the website of the National Bureau of Statistics, China is divided into three major economic regions—the eastern region, the central region and the western region—according to its location characteristics and economic development, and the regression results were obtained as shown in Table 6.

From the results in Table 6, the coefficient of the impact of the transfer of high energy-consuming industries on environmental pollution in eastern China and central China is negative, which indicates that most of these regions are the areas of transfer out, and that the reduction of high energy-consuming industries has rationalised the industrial structure and reduced the level of environmental pollution. In contrast, the coefficient for western China is positive, stating that the majority of these regions are the areas of transfer in and that the concentration of high energy-consuming industries has increased environmental pollution. It is consistent with the actual expectation that these results are attributed to the fact that eastern and central China are gradually eliminating highly polluting backward industries to upgrade and modernise them, while the western regions are largely limited in economic development level and compelled to introduce high energy-consuming industries for industrial development. The intensity of environmental regulation has a negative effect on environmental pollution in eastern China, indicating that environmental regulation measures in the eastern region can alleviate environmental pollution to a certain extent. Since the more developed regions are relatively well equipped in terms of technology and environmental protection measures, and have good conditions for policy implementation, the positive effect of environmental regulation seems more obvious. In contrast, the coefficient of that in the central and western regions is positive but none of the effects is significant, indicating that the effect of environmental regulation measures on environmental pollution is not yet significant. Furthermore, the level of environmental pollution is still on the rise due to the relatively low economic level of the central and western regions and the fact that local governments have a higher demand for economic development than for environmental quality. In terms of the interaction term, the coefficient for eastern China is significantly negative, indicating that the combination of industrial relocation and environmental regulation suppresses environmental pollution. However, the pollution-reducing effect of environmental regulations is not significant in the process of clustering high energy-consuming industries in the central and western regions. This is mainly because of the strict environmental regulations and excessive costs of pollution in the eastern region, which encourage high energy-consuming industries to shift out of the region. In comparison, the economic benefits brought by the agglomeration of high pollution industries in the western region outweigh the increased expenditure on pollution control and new technological innovation due to the strict environmental regulations, which make it easier for enterprises to pollute the environment. Environmental regulations have not played a positive incentive role, making the pollution agglomeration effect of high energy-consuming industries significant in less-developed regions.

In terms of control variables, the primary coefficient of economic development on environmental pollution is positive and the secondary coefficient is negative in eastern China, demonstrating the existence of the EKC; the relationship between economic development and environmental pollution in the central and western regions, however, does not have an inverted U-shape. The coefficients of the effect of fiscal decentralisation on the composite index of environmental pollution in eastern, central and western China are significantly negative, showing that increased fiscal expenditure leads to a reduction in environmental pollution. As the eastern region has a relatively higher degree of trade liberalisation, an optimised trade structure has a significant negative effect on environmental pollution, while the western region has a crude development of import and export trade and an inferior trade structure. Therefore, the results in Table 6 showed that the effect of OPT on environmental pollution is a significant negative effect in the eastern region while it has a negative effect in the western region. The coefficient for TRA is estimated to be positive in both the eastern and central regions while negative in the western regions. The high density of roads and railways in the eastern and central regions not only worsens atmospheric pollution during operation but also induces environmental pollution through massive pollutant emissions from construction. HUM is negatively correlated with environmental pollution, and the coefficient is greater in the eastern region than in the central and western regions, indicating that increasing the quantity of high-quality labour can alleviate the pollution problem in the region to a greater extent. This is because the eastern provinces, represented by Jiangsu, Guangdong and Shandong, have concentrated most of the resources of higher education institutions and at the same time launched a large number of policies to introduce talents. Meanwhile, the central and western regions have scarcer higher education resources and institutions—especially priority universities—and suffer from brain drain. As a result, the quantity and quality of the labour force in the eastern region are significantly higher than that in the western region, thus promoting the development of high-tech industries and improving the quality of the environment.

## 5. Conclusions

This paper started with the mechanism of the role of environmental regulations in promoting the structural adjustment of high energy-consuming industries. It then used a dynamic game model to analyse the role of different environmental regulation choices on the market concentration and the regional transfer of energy-intensive industries, respectively. Additionally, it then tested the spatial autocorrelation of the main variables using Moran’s I of spatial autocorrelation and constructed a dynamic spatial Durbin model to empirically analyse the impact of environmental regulations and the transfer of high energy-consuming industries on environmental pollution. Finally, it conducted a sub-regional sample analysis to study regional heterogeneity. The following conclusions are drawn, according to the results of the full sample: the agglomeration of high energy-consuming industries and the intensity of environmental regulations have a positive spatial effect on the composite index of environmental pollution, thus increasing environmental pollution; the combination of the transfer of high energy-consuming industries and environmental regulations significantly reduces environmental pollution; the EKC hypothesis is verified in China; transport has a positive spatial effect on environmental pollution; and the degrees of fiscal decentralisation, trade openness and human capital harm environmental pollution, with human capital having the most significant effect. In terms of spatial spillover effects—both long-term and short-term—the transfer of high energy-consuming industries and environmental regulations have positive spatial spillover effects on environmental pollution levels and exacerbate environmental pollution in neighbouring areas; however, the long-term effects are significantly higher than the short-term effects. The impact of energy-intensive industrial agglomeration on environmental pollution shows a significant negative relationship in the east and central regions, while it shows a positive relationship in the west. Environmental regulation in the east suppresses environmental pollution, but environmental regulation in the central and western regions does not solve the pollution problem but causes the environmental situation to deteriorate; in the interaction term effect of the two, the coefficient in the east is significantly negative, while the pro-decline effect in the central and western regions is not significant. Since this paper does not consider the endogeneity of variables such as environmental regulation, which has proven to be important in practice, future expansions in this topic will be considered.

To better achieve coordinated regional development and industrial transformation and upgrading, and to promote high-quality, green and sustainable economic development, the paper puts forward the following policy recommendations.

Firstly, given that environmental regulations and the positive spatial effects of the transfer of energy-intensive industries are not conducive to curbing environmental pollution, it is necessary for the receiving areas to formulate reasonable investment-attraction policies when introducing energy-intensive industries [37]. This is to balance the economic benefits and ecological and environmental carrying capacity of the area, and to support the decontamination of production facilities and industrial structures. At the same time, when transferring high-energy-consuming industries to the transferring place, it is essential to focus on the transfer of green production technology and green products and to use technology spillover to upgrade the environmental technology level of the transferring place.

Secondly, the regulatory role of environmental regulations should be maximised to achieve the best effect of transferring energy-intensive industries to reduce environmental pollution. In addition, to attract investment and carefully select the industries to be introduced, local governments need to strengthen environmental regulations, all while taking into account local environmental management capabilities and the pollution intensity of the industries to be introduced. Moreover, economic growth, transportation, fiscal decentralisation and, especially, human capital need to be taken into account through introducing preferential policies to attract talents to settle in the region, as well as through the active introduction of high-level researchers to achieve coordinated green development between regions.

Thirdly, there are significant differences between regions in China and they deserve differentiated consideration. The eastern region needs to improve the level of regional environmental regulation, while actively exporting green technologies and tools and effectively transferring advanced emissions technologies to the central and western regions. The central and western regions should develop appropriate policy guidelines for the rational layout of high energy-consuming industries and environmental protection enterprises. They should maintain a balanced ratio of the introduction of high energy-consuming industries and enterprises with advantages in environmental protection technology.

## Figures and Tables

**Table 1:** Descriptive statistics of variables.

Variables	Description	Mean	Standard Deviation	Minimum	Maximum
*EPI*	Comprehensive index of environmental pollution	1	0.5982	0.0448	2.6155
*EIT*	Industrial transfer, expressed by the concentration of high energy-consuming industries	0.0345	0.0297	0.0014	0.1460
*lnPER*	Performance-based environmental regulation (logarithmic form)	0.4926	0.4365	0.00002	2.4966
*CER*	Cost-based environmental regulation	0.0015	0.0013	0.00008	0.0096
*lnECD*	Economic development level (logarithmic form)	10.1780	0.7199	8.1895	11.6801
*FIS*	Fiscal decentralization	0.0333	0.0166	0.0058	0.0993
*OPT*	Trade openness	0.3128	0.3605	0.0134	1.6682
*lnTRA*	Transportation (logarithmic form)	11.4293	0.8744	8.8160	12.7031
*HUM*	Human capital level	0.0162	0.0065	0.0039	0.0357

**Table 2:** Spatial correlation tests for core variables.

Year	*EPI*		*EIT*		*lnPER*		*CER*	
	Moran’s I	Z-Value	Moran’s I	Z-Value	Moran’s I	Z-Value	Moran’s I	Z-Value
2003	0.115 *	1.356	0.207 **	2.246	0.127 *	1.596	0.219 **	2.288
2004	0.123 *	1.442	0.232 ***	2.490	0.152 **	1.751	0.142 *	1.452
2005	0.121 *	1.411	0.234 ***	2.525	0.186 **	2.054	0.135 *	1.435
2006	0.124 *	1.431	0.231 ***	2.507	0.176 **	1.952	0.176 **	1.947
2007	0.122 *	1.416	0.228 ***	2.479	0.182 **	1.986	0.168 *	1.949
2008	0.142 *	1.595	0.245 ***	2.638	0.160 **	1.820	0.192 **	2.281
2009	0.152 **	1.686	0.230 ***	2.524	0.177 **	1.953	0.309 ***	3.155
2010	0.152 **	1.692	0.237 ***	2.560	0.181 **	2.025	0.333 ***	3.451
2011	0.155 **	1.731	0.248 ***	2.672	0.197 **	2.186	0.303 ***	3.051
2012	0.151 **	1.695	0.246 ***	2.666	0.212 ***	2.353	0.204 **	2.214
2013	0.144 *	1.633	0.237 ***	2.600	0.218 ***	2.455	0.310 ***	3.239
2014	0.139 *	1.578	0.239 ***	2.642	0.251 ***	2.806	0.269 ***	3.186
2015	0.133 *	1.515	0.250 ***	2.766	0.214 ***	2.420	0.200 **	2.149
2016	0.142 *	1.588	0.241 ***	2.697	0.129 *	1.589	0.190 ***	2.482

Note: *, ** and *** represent significant at the level of 10%, 5%, and 1%, respectively.

**Table 3:** Test results of model selection.

Test Method	Test Statistics Result	*p*-Value
LM-Lag	7.131	0.008
Robust LM-Lag	8.221	0.004
LM-Error	13.191	0.000
Robust LM-Error	15.281	0.000
LR-SDM-SAR	39.510	0.000
LR-SDM-SEM	36.490	0.000
Hausman	13.310	0.036

**Table 4:** Model regression results.

Variable	Dynamic Spatial Durbin Model		Static Spatial Durbin Model		General Panel Regression Model	
	(1)	(2)	(3)	(4)	(5)	(6)
*L.EPI*	0.259 *** (9.32)	——	——	——	——	——
*L.WEPI*	0.102 * (1.94)	0.463 *** (3.55)	——	——	——	——
*EIT*	0.272 *** (3.97)	0.398 *** (5.61)	0.315 ** (2.50)	0.392 * (1.90)	0.828 * (1.83)	0.228 * (1.72)
*lnPER*	0.017 *** (9.04)	——	0.023 *** (12.47)	——	0.025 *** (3.97)	——
*CER*	——	0.011 *** (3.86)	——	0.006 *** (3.58)	——	1.513 (0.88)
*lnECD*	0.071 *** (3.95)	0.028 (0.38)	0.090 *** (5.13)	0.063 (1.59)	0.084 ** (2.44)	0.047 (0.61)
*lnECD^2^*	−0.002 ** (−2.37)	−0.001 (−0.18)	−0.003 *** (−3.56)	−0.001 (−0.95)	−0.004 * (−1.83)	−0.002 (−0.56)
EIT*PER	−1.053 *** (−17.68)	——	−1.179 *** (−20.69)	——	−1.176 *** (−7.17)	——
EIT*CER	——	−0.011 ** (−1.99)	——	−0.022 (−0.55)	——	2.031 (0.32)
*FIS*	−0.039 (−0.35)	−0.560 ** (−2.24)	−0.073 (−0.66)	−0.096 (−0.39)	0.170 (0.96)	0.407 (0.74)
*OPT*	−0.012 ** (−2.03)	0.001 (0.08)	−0.006 (−1.57)	0.011 (1.18)	−0.003 (−0.51)	−0.005 (−0.42)
*lnTRA*	0.014 *** (5.42)	0.035 *** (8.96)	0.020 *** (7.53)	0.012 * (1.90)	0.015 *** (5.35)	0.008 (1.27)
*HUM*	−0.645 ** (−2.46)	−3.032 *** (−8.03)	−0.578 * (−1.72)	−1.798 *** (−3.42)	−0.397 (−1.36)	−1.662 ** (−2.13)
W*EIT	0.588 *** (3.55)	−0.103 (−0.10)	0.801 *** (5.00)	0.113 (0.17)	——	——
W*PER	0.008 * (1.85)	——	0.002 (0.52)	——	——	——
W*CER	——	0.008 (1.12)	——	0.006 (1.45)	——	——
W*EIT*PER	−0.378 * (−1.92)	——	−0.632 *** (−4.35)	——	——	——
W*EIT*CER	——	−0.010 (−0.07)	——	−0.191 * (−1.84)	——	——
*Spatial ρ*	0.337 *** (5.25)	0.245 *** (3.45)	0.175 * (1.73)	0.136 * (1.78)	——	——
*R^2^*	0.789	0.685	0.165	0.081	0.784	0.652
*Log-likelihood*	199.546	111.446	109.546	109.546	——	——
*N*	390	390	420	420	420	420

Note: *, ** and *** mean *p* < 0.1, *p* < 0.5, *p* < 0.01, respectively. The values in parentheses are the values of the corresponding coefficients of the t statistic values.

**Table 5:** Spatial effect decomposition results of the model.

Variable	Dynamic Spatial Durbin Model				Static Spatial Durbin Model	
	Short-Term Effect		Long-Term Effect		Long-Term Effect	
	Direct Effect	Indirect Effect	Direct Effect	Indirect Effect	Direct Effect	Indirect Effect
*EIT*	0.240 *** (3.61)	0.654 ** (2.01)	0.285 ** (2.34)	1.044 * (1.78)	0.335 *** (4.54)	0.965 *** (3.85)
*lnPER*	0.016 *** (8.82)	0.013 ** (2.41)	0.020 *** (8.54)	0.023 *** (3.98)	0.019 *** (13.61)	0.017 *** (3.98)
EIT*PER	−1.042 *** (−16.89)	−0.229 * (−1.69)	1.420 *** (15.84)	0.036 * (1.82)	1.159 *** (20.26)	−0.338 * (−1.92)
*EIT*	0.810 (1.54)	−0.145 (−0.14)	0.842 (1.56)	−0.430 (−0.22)	0.476 (1.35)	−0.088 (−0.12)
*CER*	0.012 *** (4.09)	0.009 (1.09)	0.013 *** (4.25)	0.025 (1.60)	0.006 *** (3.69)	0.007 (1.61)
EIT*CER	−0.005 (−0.07)	−0.017 (−0.12)	−0.007 (−0.10)	−0.034 (−0.13)	−0.027 (−0.65)	−0.212 * (−1.75)

Note: *, ** and *** mean *p* < 0.1, *p* < 0.05, *p* < 0.01, respectively. The values in parentheses are the values of the corresponding coefficients of the t statistic values.

**Table 6:** Regression results of regional heterogeneity.

Variable	Eastern Region		Central Region		Western Region	
	(1)	(2)	(3)	(4)	(5)	(6)
*L.EPI*	——	——	——	1.005 *** (13.65)	0.116 * (1.89)	——
*L.WEPI*	0.019 (0.37)	0.360 ** (2.22)	0.271 * (1.89)	——	−0.113 (−1.25)	−0.720 *** (−3.35)
*EIT*	−0.280 ** (−2.02)	−0.198 *** (−2.99)	−0.887 *** (−3.52)	−1.155 *** (−3.97)	1.394 (1.69)	0.405 (0.57)
*lnPER*	−0.029 ** (−2.12)	——	0.002 (1.14)	——	0.022 (0.72)	——
*CER*	——	−2.436 *** (−4.16)	——	−1.828 * (−1.75)	——	0.009 ** (2.22)
*lnECD*	0.114 * (1.79)	0.239 *** (3.31)	0.035 (1.23)	0.038 (0.52)	−0.002 (−0.04)	−0.249 * (−1.83)
*lnECD^2^*	−0.004 ** (−2.43)	−0.011 ** (−2.15)	−0.002 (−1.03)	−0.004 (−0.85)	0.002 (0.63)	0.008 (1.63)
EIT*PER	−1.048 *** (−6.42)	——	1.400 *** (7.64)	——	3.717 *** (12.08)	——
EIT*CER	——	−0.089 (−1.44)	——	1.930 ** (2.41)	——	0.009 (0.05)
*FIS*	−0.106 (−0.63)	−1.032 *** (−4.40)	−0.614 * (−1.90)	−0.139 *** (−4.67)	−0.456 ** (−2.04)	−0.066 (−0.15)
*OPT*	−0.036 * (−1.87)	−0.025 * (−1.72)	−0.052 * (−1.82)	−0.015 (−0.48)	0.025 ** (2.43)	0.045 * (1.92)
*lnTRA*	0.016 *** (3.88)	0.044 *** (8.19)	0.003 (0.59)	0.153 * (1.82)	−0.003 (−1.18)	−0.026 ** (−2.48)
*HUM*	−0.535 *** (−3.20)	−1.411 * (−1.91)	−0.112 (−0.23)	−0.025 (−1.57)	−0.678 * (−1.92)	0.299 (0.61)
*Spatial ρ*	0.394 *** (3.23)	0.322 *** (3.56)	0.352 *** (3.45)	0.295 ** (2.24)	0.336 *** (3.23)	0.316 * (1.88)
*R^2^*	0.887	0.867	0.529	0.6916	0.9308	0.674
*Log-likelihood*	106.295	178.026	512.385	202.400	521.664	61.747
*N*	156	156	117	117	117	117
Individual effect	Fixed	——	Fixed	Fixed	Fixed	Fixed
Time effect	——	Fixed	——	Fixed	——	Fixed

Note: *, ** and *** mean *p* < 0.1, *p* < 0.05, *p* < 0.01, respectively. The values in parentheses are the values of the corresponding coefficients of the t statistic values.

## Data Availability

The data presented in this study are available on request from the corresponding author.
